# Value of Diaphragm Ultrasonography for Extubation: A Single-Blinded Randomized Clinical Trial

**DOI:** 10.1155/2023/8403971

**Published:** 2023-09-19

**Authors:** T. G. Toledo, M. R. Bacci

**Affiliations:** Centro Universitario FMABC, Santo Andre, Brazil

## Abstract

**Introduction:**

Daily evaluation of mechanically ventilated (MV) patients is essential for successful extubation. Proper withdrawal prevents complications and reduces the cost of hospitalization in the intensive care unit (ICU). Diaphragm ultrasonography (DUS) has emerged as a potential instrument for determining whether a patient is ready to be extubated. This study compared the efficacy rate of extubation using a standard withdrawal protocol and DUS in patients with MV.

**Methods:**

A randomized, parallel, single-blind, controlled study was conducted on ICU patients undergoing MV. Patients were randomly assigned to either the control (conventional weaning protocol) group or intervention (DUS-guided weaning) group in a 1 : 1 ratio. The primary outcome measure was the rate of reintubation and hospital mortality.

**Results:**

Forty patients were randomized to the trial. The mean age of the sample was 70 years, representing an older population. The extubation success rate was 90% in both groups. There was no reintubation in the first 48 hours and only two reintubations in both groups between the second and seventh days. The hospital mortality risk in patients with acute kidney injury was positively correlated with age and the need for hemodialysis. *Discussion*. This study demonstrates the usefulness of DUS measurement protocols for withdrawing MV. The rate of reintubation was low for both cessation methods. As a parameter, the diaphragm thickness fraction comprehensively evaluates the diaphragm function. The results demonstrate that DUS has the potential to serve as a noninvasive tool for guiding extubation decisions. In conclusion, using DUS in patients with respiratory failure revealed no difference in reintubation rates or mortality compared with the conventional method. Future research should concentrate on larger, multicentered, randomized trials employing a multimodal strategy that combines diaphragmatic parameters with traditional clinical withdrawal indices.

## 1. Introduction

The daily assessment of mechanical ventilation (MV) patients is essential to successfully withdraw ventilatory support [1]. Early extubation, if mishandled, can result in clinical complications and inflammatory stress [1]. On the other hand, the delay of MV withdrawal might result in ventilation-associated pneumonia and diaphragm atrophy [2]. Ultrasonography introduction for diaphragm analysis started in 1975 [3], and since then, diaphragm mobility has been studied as a target for MV withdrawal [4–6]. To this end, medical institutions have started to develop clinical guidelines for timely weaning from MV and avoiding reintubations, which would increase patient morbidity and mortality [7–9]. Patients with MV frequently have a higher length of stay (LOS) and, consequently, an increase in in-hospital intensive care unit (ICU) costs. Recently, during the COVID-19 outbreak in the United States, a study showed that the LOS and costs of MV COVID-19 patients were higher (16 days and $47,454 on average) [10]. Weaning from MV is mainly conducted by clinical judgement, evaluating Tobin's index and respiratory parameters from the ventilation machine. In 2010, Bouhemad suggested the creation of an ultrasonographic score to assess the measurement of the diaphragm thickness and mobility during the respiratory cycle [11]. However, the studies are still heterogeneous and have a small sample, compromising their external validity. To this end, this study aimed to compare the extubation success rate in MV patients using a standard withdrawal protocol and the ultrasonography evaluation of the diaphragm (DUS).

## 2. Methods

### 2.1. Study Design

This was a randomized, parallel-controlled study, single-blinded, in which 40 patients undergoing mechanical ventilation in an intensive care unit were enrolled. A convenience sample was chosen for this evaluation as more data are required regarding a gold standard method for weaning from mechanical ventilation. Enrollment was made sequentially according to the inclusion criteria.

A simple randomization method was chosen due to the sample size through the website https://www.sealedenvelope.com/simple-randomiser/v1/lists. Forty sealed envelopes containing the allocation group in a 1 : 1 ratio were made, and an independent staff team performed the picks and delivered them to the investigator. All efforts were made to avoid bias after the reveal of which group they were allocated to. Patients were randomized into two groups with twenty individuals each as follows:

- *Control Group.* Weaning from MV according to the hospital standard of care protocol
- *Intervention Group*. Weaning from MV with ultrasound diaphragm measurement

The standard of care for the local MV weaning protocol consisted of patients with the following characteristics:

- Presence of spontaneous ventilation mode for more than 6 hours
- Evidence of low quantity of pulmonary secretion
- Heart rate <140 beats per minute (bpm)
- No signs of respiratory distress
- Stable consciousness status was evaluated by one of the following methods: RASS result of 0 or 1, SAS sedation scale result between 3 and 5, or Glasgow Coma Scale >7
- Existence of arterial pH > 7.34 and arterial partial pressure of oxygen with a fraction of inspired oxygen (PaO2/FIO2) ratio >200 and an arterial partial pressure of oxygen >59 mmHg with an inspired oxygen fraction <40%;
- Evidence of a driving pressure (DP) <15 cmH_2_O
- Evidence of positive end-expiratory pressure (PEEP) <8 cmH_2_O
- Existence of a fluid balance result inferior to 1000 ml in the previous 24 h
- Hemodynamically stable, defined by a mean arterial pressure of at least 65 mmHg or with a low-dose use of a vasoactive drug regimen

The intervention group used the same parameters to qualify for weaning and withdrawal from MV by adding the DUS criteria. The DUS parameter that indicated weaning from MV was the diaphragm thickness fraction (DTF), with a delta result greater than 30%. DTF is the difference between the thickness of the diaphragm at the end of inspiration and the end of expiration. DTF accounts for diaphragm thickness and contractility, offering a more comprehensive assessment of diaphragm function. The calculation was performed after five complete respiratory cycles for each patient as soon as they were placed on spontaneous mode ventilation. All measurements were made by the same examiner using the Mindray Resona 7 device in M mode with a linear transducer and subcostal window in patients resting at a forty-five degree angle.

### 2.2. Study Population

Adult patients with respiratory failure requiring MV in the intensive care unit were sequentially included after consent in this trial. The exclusion criteria involved patients with neuromuscular disease, extensive subcutaneous emphysema, MV for more than 14 days, tracheostomy, and patients in the postoperative period with pleural space opening. The primary outcome of this study was the rate of reintubation after MV withdrawal and death during hospitalization.

### 2.3. Statistical Analysis

Microsoft Excel 2013 was used to elaborate the database and the SPSS v 21.0 program for statistical analysis in the statistical analysis method. The Shapiro–Wilk test was used to verify the data distribution as a function of sample size. According to the data distribution, descriptive statistics used means, standard deviations, medians, and percentiles for quantitative and qualitative variables. Comparisons between groups were performed using Student's *t*-tests for independent samples, Mann–Whitney, chi-square, and Fisher's exact tests. A significance level of 0.05 (5%) was defined for this study. The binary logistic regression analysis evaluated the predictive variables for the occurrence of death during the hospitalization period. The Institution Review Board approved the protocol with the following registration number: 3.345.462. All participants consented before they participated in the study. The CONSORT checklist for randomized clinical trials was used in this study.

## 3. Results

### 3.1. Sample Characteristics and Ventilatory Parameters

The baseline characteristics of the sample are shown in Table 1. 40 participants had a median age of 70 years, and 60% were female. The primary reason for MV was respiratory failure, with the predominance of clinical diagnosis at admission over surgical cases. Serum CRP was predominantly high in both groups. The oxygen partial pressure had a median of 92.5 mmHg in the US group and 87.5 mmHg in the control group, with a median PEEP of 6.5 cmH_2_O and 7 cmH_2_O, respectively. The severity of lung disease was evaluated by the FiO2/PaO2 ratio. Results ranged from 381.77 for the US group to 363.15 for the control group (Table 2). Both groups also had a similar occurrence of vasopressor administration during mechanical ventilation weaning.

### 3.2. Reintubation and the Chance of Death

There was no difference between groups regarding reintubation with an interval of less than 48 h (Table 3), as there were no reintubations in this period. The occurrence of reintubation between the second and seventh days after extubation was similar; each group had two reintubations. The two groups had the same number of reintubations. Both groups were composed primarily of clinical diagnoses at the unit's admission.

A logistic regression model was built to verify the variables related to the chance of death during hospitalization (Table 4). All variables in Table 1 were tested to build the final model. Of the variables tested, only age and the need for hemodialysis in acute kidney injury patients demonstrated a correlation. The model containing age and need for hemodialysis was significant (*X*^2^(^2^) = 14.68, *p*=0.001, and *R*^2^_Nagelkerke_ = 0.415.

Spearman's correlation was performed to analyze the relationship between positive pressure above PEEP and the diaphragmatic ultrasound measurement relationship. The test showed an inverse relationship (*p*=0.037) between the studied variables.

## 4. Discussion

In this single-center, single-blinded, randomized controlled trial, we demonstrated the utility of a DUS measurement protocol for withdrawal in MV patients. We found a similar success rate between standard and ultrasound weaning protocols in the majority of the elderly population.

Mechanical ventilation is vital in managing critically ill patients with respiratory failure. However, prolonged mechanical ventilation is associated with various complications, including ventilator-induced diaphragm dysfunction (VIDD), ventilator-associated pneumonia, and barotrauma [12, 13]. Extubation in critically ill patients is often full of risk and conjecture for the multidisciplinary team. Traditionally, clinical methods for MV weaning involve the use of various parameters, such as rapid shallow breathing index (RSBI), maximal inspiratory pressure (MIP), and tidal volume [12, 13]. However, these methods have limitations. The search for more accurate, noninvasive, and patient-specific tools for MV withdrawal has led to the emergence of DUS as a potential alternative [3, 8, 14–16]. In our study, we found a low rate of reintubation, which is consistent with the precise moment to indicate MV withdrawal in both methods.

Several diaphragmatic parameters have been proposed and investigated, including diaphragm thickness, diaphragm excursion, and diaphragm thickening fraction (DTF) [8, 17–19]. B-mode ultrasonography is used to evaluate diaphragm thickness at the zone of apposition, and changes in thickness over time have been linked to diaphragm atrophy or hypertrophy. On the other hand, diaphragm excursion uses M-mode ultrasonography to assess the movement of the diaphragm during inspiration and expiration, with decreased excursion suggesting worse diaphragm function [8, 20]. We utilized DTF as the best parameter in our study using M-mode. According to recent research, DTF may hold substantial potential as a reliable predictor of extubation success [14]. DTF was proven to be an accurate predictor of extubation success in the research by Ferrari et al., with a cutoff value of 30% producing good sensitivity and specificity [21].

Creating guidelines and machine learning in medicine is no longer so innovative. The regularity of the results expected with this is similar to that expected when using a numerical value that validates the procedure's success [19]. A similar and promising study that used the US of the diaphragm in mechanical ventilation weaning in patients with COPD compared the success of extubation using data from the diaphragm after 5 and 30 minutes of spontaneous breathing tests [22]. The idea of creating a strategy in which a low-cost, risk-free, bedside examination can be used to validate the individual's muscle competence to ensure their spontaneous breathing is extremely interesting. Elderly patients, on the other hand, have a lower muscle density, which necessitates adopting such techniques.

Elderly patients requiring mechanical ventilation are at an increased risk of mortality due to their age-related decline in physiological reserves and a higher likelihood of presenting with multiple comorbidities [23]. Furthermore, acute kidney injury (AKI) is a common and severe complication in this population, significantly exacerbating the mortality risk among elderly patients on mechanical ventilation [24]. In our study, we also had an elderly population and a positive correlation with mortality when AKI and hemodialysis were present in these intubated patients. Several studies have shown that AKI has a negative impact on the outcomes of elderly patients on mechanical ventilation. Gong et al. observed in a prospective cohort research study that the development of AKI in elderly patients was related to a substantially increased death rate, even after controlling for relevant confounders [25]. Another study by Hoste et al. found that AKI significantly increased the risk of hospital mortality and lengthened the duration of mechanical ventilation and ICU stay in critically older persons [26]. Given this increased mortality risk, identifying and managing modifiable risk factors for AKI, such as improving fluid balance, avoiding nephrotoxic exposure, and continuously monitoring renal function, is critical. Early detection and management may improve patient outcomes and lower mortality risk in this vulnerable group.

There are some limitations to this trial. It was conducted at the beginning of the COVID-19's first wave in Brazil, which limited the inclusion of other sites and a larger sample. The sample number limitation might have caused the failure to reject the null hypothesis, even in the context of a convenience sample. All the efforts were made to reduce the risk of randomization flaws due to the nature of this trial, a single-center study. The investigator did not control the inclusion of patients regarding the cause of intubation, as they were sequentially included and randomized, which could have caused an imbalance between groups. Nevertheless, the study showed the harmless use of a point-of-care device to guide physicians through mechanical ventilation weaning. The DTF measurement is operator-dependent, and to avoid such bias, the same examiner made all evaluations.

In conclusion, using an ultrasound diaphragm measurement protocol in respiratory failure patients showed no difference in the reintubation rate or mortality compared to the traditional method. Future directions should guide targeted disease ultrasound weaning protocols in multicenter, larger randomized trials, incorporating a multimodal approach with multiple diaphragmatic parameters and traditional clinical weaning indices.

## Figures and Tables

**Table 1:** Demographics and baseline characteristics.

US (*n* = 20)					Control (*n* = 20)			*p*
	Median	Percentile			Median	Percentile		
		25		75		25	75	
Age	78	59		80.8	67	55.3	76	0.244^*∗*^
BMI	26	22		30	24	22	28	0.636^*∗*^
Female	14 (70%)				10 (50%)			0.167^*τ*^

*Admission diagnosis*								
Surgical	7 (35%)				6 (30%)			0.500^*τ*^
Clinical	13 (65%)				14 (70%)			

*Intubation reason*								
Coma	5 (25%)				10 (50%)			0.095^*τ*^
Respiratory failure	15 (75%)				10 (50%)			

*Presence of infection*								
No infection	1 (5%)				4 (20%)			0.357^●^
Infection only	13 (65%)				11 (55%)			
Sepsis	6 (30%)				5 (25%)			

pH	7.45	7.42		7.47	7.44	7.40	7.48	0.465^◊^

pCO_2_ (mmHg)	38	35.9		45	37.6	32	41	0.223^*∗*^

pO_2_ (mmHg)	92.5	80.1		109.8	87.5	68.5	109	0.298^*∗*^

MAP (mmHg)	88 ± 14				91 ± 14			0.590^◊^

Fluid balance (mL)	315	−468	770		480	−265	988	0.379^*∗*^

*D vitamin*								
No result	3 (15%)				5 (25%)			0.235^●^
Low	8 (40%)				11 (55%)			
Normal	9 (45%)				4 (20%)			

*Atrial natriuretic peptide*								
No result	3 (15%)				5 (25%)			0,537^*τ*^
High	9 (45%)				10 (50%)			
Normal	8 (40%)				5 (25%)			

*CRP*								
Normal	4 (20%)				4 (20%)			0,653^*τ*^
High	16 (80%)				16 (80%)			

^*∗*^Mann–Whitney; ^*τ*^Fisher; ^●^Chi-square, ^◊^Student's *t*-test; ^*∗*^Mann–Whitney test; ultrasound group (US); mean arterial pressure (MAP); C-reactive protein (CRP).

**Table 2:** Ventilatory parameter comparison between groups.

	US median	P25	P75	Control median	P25	P75	*p*
Respiratory rate	16	15	18	16	12	18	0.303^*∗*^
FiO_2_ (%)	25	25	29	25	21	30	0.885^*∗*^
Time in PSV mode (h)	10	6	26	17	6	26	0.880^*∗*^
FiO_2_/PaO_2_	381.77 ± 118.51			363.15 ± 125.41			0.632^◊^
PEEP (cmH_2_O)	6.5	6	8	7	6	7	0.921^*∗*^
Positive pressure above PEEP (cmH_2_O)	8	7	11.5	9	8	10,75	0.621^*∗*^

*Vasopressor use at extubation*							
Yes	6 (30%)			5 (25%)			0,500^*τ*^
No	14 (70%)			15 (75%)			

^*∗*^Mann–Whitney test; ^◊^Student's *t*-test; ^*τ*^Fisher's exact test.

**Table 3:** Reintubation comparison between 48 h and 168 h evaluation time and death among groups.

	US	Control	*p*
*Reintubation*			
Yes	2 (10%)	2 (10%)	0.598^●^
No	18 (90%)	17 (85%)	
Death	9 (45%)	7 (35%)	0,374^*τ*^

^●^Chi-square test; ultrasound group (US); ^*τ*^Fisher's exact test.

**Table 4:** Logistic regression model to risk of death during hospitalization.

Predictive factors	B	OR (95% CI)	*p*
Constant	−8.375		
Age	0.105	1.111 (1.03−1.199)	0.001
Hemodialysis	2.165	8.714 (1.008–75.358)	0.049

Logistic regression constant (B).

## Data Availability

The structured data used to support the findings of this study are included within the article.
